# Local Efficacy of Corticosteroids as an Adjuvant for Periarticular Cocktail Injection in Simultaneous Bilateral Total Knee Arthroplasty: A Prospective Randomized Double-Blind Controlled Trial

**DOI:** 10.1155/2021/5595095

**Published:** 2021-05-19

**Authors:** Huiming Peng, Wei Wang, Jin Lin, Xisheng Weng, Wenwei Qian, Wenda Wang

**Affiliations:** Department of Orthopaedic Surgery, Peking Union Medical College Hospital, Chinese Academy of Medical Sciences & Peking Union Medical College, Beijing 100730, China

## Abstract

Multimodal cocktail periarticular injections comprising corticosteroids are the most suggested therapy for postoperative discomfort and swelling following total knee arthroplasty (TKA). Nevertheless, previous findings cannot be applied to instances of unilateral total knee arthroplasty on bilateral knees. This randomized, prospective, double-blind, controlled clinical study examines the efficacy as well as safety of periarticular multimodal cocktail injection along or sans corticosteroids in certain situations. The 60 patients (120 knees) that experienced concurrent bilateral total knee arthroplasty were provided periarticular injections along additional betamethasone (7 mg) in the randomized knee, as well as the other knee, where corticosteroid was not administered. Key results were “pain scores at rest as well as in action” on a visual analogue scale of 11 pt. Other results included motion range, swelling of the thigh, Hospital for Special Surgery score (HSS score), and adverse effects were measured between the two sides. No statistically promising variations were found in the visual analogue scale ranking, motion range, girth of the thigh, and HSS score, as well as complications between the two sides. The impact on treatment outcomes was maintained between the knees on postoperative day 3 or at 3 months of follow-up. Multimodal periarticular injection without corticosteroid will alleviate postoperative swelling and pain. More studies are needed for the use of betamethasone as a corticosteroid in periarticular multimodal cocktail injections. This Chinese Clinical Trial Registry is registered with ChiCTR-OPC-17013503, dated 2017-11-23, available from http://www.chictr.org.cn/showproj.aspxproj=23146.

## 1. Introduction

Total knee arthroplasty is a successful alternative for the end-stage joint situation [1] yet still is one of the most unpleasant [2, 3] and disgruntled interventions [4]. The magnitude of acute postsurgery pain is also indicative of slow recovery [5, 6] and adverse effects [7]. Contrarily, when the pain is adequately handled, it may promote early recovery, minimize the duration of stay, increase patient comfort, and can promote opioid-free therapy [8, 9]. Over the last decades, almost all protocols often recommended an amalgamation of pain relief strategies before, following, and succeeding surgery [3, 10–13]. The strategy tends to be rational, since most situations involve concomitant treatment to treat extreme symptoms and preexisting nervous system sensitization or to avoid adverse events.

Corticosteroids are amongst the oldest anti-inflammatory agents for the management of arthritis of the knee and other joints [14]. Owing to its ability to control inflammation, this family of drugs can also be used for postoperative treatment. Some previous findings have seen favourable effects of corticosteroid [14–18], whereas others have observed inconsistent or poor consequences [19–21].

The treatment of patients seeking total knee arthroplasty on both sides is much more ambiguous. The new epidemiological reports estimate that up to 42% of people experiencing knee pain will require surgery on either knee [22]. Both techniques may be carried out at the same time or one at a time. Kwon and colleagues [23] found significant positive and very short-lived effects of corticosteroids in the bilateral complete arthroplasty of the knee. In the report, the enrolled patients had endured two different surgical cases, one on each leg, including at least three months separating each.

Bilateral arthroplasty of the knee may tend technically to be a transient process [24, 25] but is also linked to poor results for the second knee surgery [26]. Hyperalgesia during the duration of the subsequent surgery [27, 28] and perceptions associated with discomfort [29] could be accountable for the excessive encounter. Concomitant treatment restricts surgery to a single event [30], lowers hospitalization time and costs, reduces time and costs associated with hospitalization [31], encourages the symmetrical improvement of function [32], promotes the symmetrical change of function, and increases satisfaction [33]. It is reasonable to believe that patients requiring surgery on both knees can have greater severity of knee-related pain and swelling than expected postoperative pain. Therefore, findings from unilateral trials may not be applied explicitly to bilateral events. Since then, there have been a small number of studies presenting bilateral complete knee arthroplasty, although probably no overlapping cases have been reported.

To answer this, we questioned whether the introduction of corticosteroid to the multimodal periarticular combination affords some improved pain relief, functional improvement, and tolerability in patients receiving concurrent bilateral complete knee arthroplasty.

## 2. Materials and Methods

### 2.1. Study Design and Setting

The research was a double-blind, single-centre, prospective, randomized, and controlled trial. The Institutional Review Board provided due permission to commence the trial. Duly endorsed informed consent was collected from the patient before surgery, which indicated the patient's volunteer participation in the study.

### 2.2. Participant Recruitment

Patients eligible for unilateral total knee arthroplasty, bilateral total knee arthroplasty, or reconstruction of total knee arthroplasty were exempted. Besides, patients with comorbidities of either of the medications in the sample with a history of hypersensitivity, renal disorders, antiquity of cardiac disorders, thrombotic disorders, or knee joint intervention were exempted from this study. Osteoarthritis was observed to be the presurgical diagnosis in all of the cases. The medical experts who participated in the study were unaware of the treatment during the study.

### 2.3. Sample Size Calculation

A decrement of 20 points on the scale of the pain was documented to be scientifically significant [34]. In time for enabling a drop-out rate of 20 percent (8 participants), 51 patients in each group were supposed to identify the major mean variations (and SD) of 20 ± 33 points on the scale of pain with a two-sided significance of 5% and power of 80%.

### 2.4. Interventions

#### 2.4.1. Surgical Procedures

In the surgery room instantaneously preoperatively, either of the knees was assigned to obtain a multimodal periarticular cocktail injection in the presence and absence of steroids by use of envelope method. Prior to combining the cocktail injection, the circulating nurse selected the envelope as well, as the patient and other staffs were unaware of the implications of the intervention. The knee assigned as control received 60 mL multimodal cocktail periarticular injection and ropivacaine 200 mg/20 mL (AstraZeneca AB, Sweden), adrenaline 0.25 mg (1 : 1000), flurbiprofen axetil injection 50 mg/5 mL (Beijing Tide Pharmaceutical Co., Ltd., China), tranexamic acid 2000 mg/20 mL, and morphine 10 mg/1 mL (Guangzhou Baiyunshan Pharmaceutical Co., Ltd., China), and 14 mL of normal saline solution was prepared in three 20 mL syringes. In the knee receiving the intervention, the 60 mL multimodal cocktail periarticular injection was similar to that of the control knee, with the exception of the addition of betamethasone 7 mg/1 mL (Schering-Plough Labo N.V., Belgium). The first 20 mL of the cocktail was infused instantly prior to implanting the prosthesis into the posterior surface of the capsule as well as the frameworks of the knee joint. After the implant process was complete, the rest of the 40 mL of the combination was made to infuse at extension framework, synovial membrane, pes anserinus, anterior capsule, periosteum, iliotibial band, calcaneal, and collateral connective tissue. Each of the total knee arthroplasties was conducted underneath a 250 mm Hg tourniquet control by a chief orthopaedist (JL). The protocols associated with medial parapatellar were used for each knee and either G-II PS (Smith & Nephew, Memphis, USA) or NexGen PS (Zimmer, Limerick, Ireland) was embedded. The well-planned and validated rehabilitation procedures have been enacted for the preoperative period. It included the series of motion exercises beginning on the first day, along with ample rehabilitation getting accomplished either in the patient room or in the physiotherapy centre till discharge. Postsurgery thermal and chemical thromboprophylaxis was established for volunteers and was comprised of thromboembolic deterrent stockings alongside daily low-molecular-weight heparin subcutaneous injection till discharge. Wounds were drained under a vacuum. Cefuroxime (1.5 g, GlaxoSmithKline, UK) was provided preoperatively as well as every 12 h during the first 24 h postoperative period.

### 2.5. Multimodal Pain Management Protocol

Patients were administered with 40 mg parecoxib (Dynastat, Pfizer, USA) diluted to 5 mL with 0.9% normal saline every 12 h after operation for about 3 days followed by an oral nonsteroidal anti-inflammatory drug (60 mg of Loxoprofen Sodium Tablets Tid, Daiichi Sankyo, Japan). Patient-controlled analgesia (1 mg morphine/press) was used in each case for 3 days after surgery. There was no precedent or morphine loading injection. Where applicable, aminophenol oxycodone (Mallinckrodt Inc., USA) had been used as emergency analgesics.

### 2.6. Outcome Measurements

#### 2.6.1. Primary Outcome

The observations were conducted by the staff members who were unaware of the type of therapeutic interventions provided to the patients. A visual analogue scale was used to rate the pain intensity where 0 mm demonstrated the condition of “no pain” and 100 mm demonstrated the condition of “extreme pain.” The lapse where the patient was awake while transferring postanaesthesia care unit was termed as time “zero.” The score of pain during resting phase (“preoperatively, time zero, and postoperatively 6 h, 12 h, 24 h, day 2, and day 3”) and the score of pain during movements (“postoperative 6 h, 12 h, 24 h, day 2, and day 3”) were taken into consideration.

### 2.7. Secondary Outcomes

**Figure 1:**
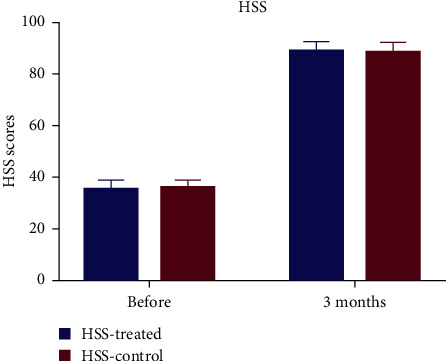
Knee HSS (Hospital for Special Surgery) score before and 3 months after total knee arthroplasty.

The secondary outcomes include the following:

- Range of movement of the knee throughout physiotherapy (“preoperatively and postoperatively on days 1, 3, and 5, as well as 3 months”). Range of motion (ROM) was used by two methods: the Hospital for Special Surgery scale and the goniometer. The Hospital for Special Surgery (HSS) scale is a subjective scores standardized questionnaire (Figure 1), while the goniometer is more independent of participant's reporting. For the latter, a full extension of the knee joint is defined as 0°, and the angle of knee hyperextension is defined as a negative angle; the angle of knee flexion minus the angle of extension is calculated to obtain the degree of ROM of the knee joint.
- The perturbance of the thighs (“two points: the thickness of the thigh at the posterior edge of the patella and the proximal girth at 5 cm from the upper border of the patella; preoperative and postoperative days 1, 2, and 3”).
- Clinical ranking (“preoperatively and postoperatively at 3 months”; thigh surface temperatures on either leg (“5 cm proximal from the upper boundary of patella; preoperatively and postoperatively at days 1, 2, and 3”) and adverse events during the trial.

### 2.8. Statistical Analysis

The Statistical Package for Social Sciences (SPSS Inc, IBM, version 22) was used for statistical analysis. Statistical guidance was obtained prior to preparing the report. For continuous variables, the outcome is described as the mean and range and was correlated among groups by the use of Student's *t*-test. In the case of categorical variables, the values were organized as frequency and proportion.

## 3. Results

**Figure 2:**
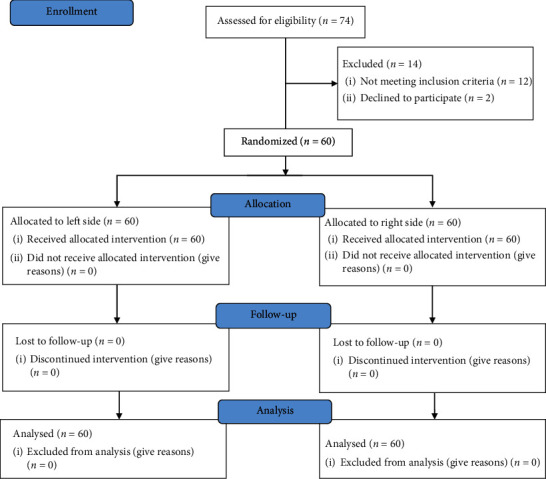
CONSORT flow diagram showing the flow of patients through each stage of the trial.

The study included enrolment of patients who were expected to undergo simultaneous bilateral total knee arthroplasties along with rheumatoid arthritis or osteoarthritis commencing from July 2017 to July 2018 i n the study clinical setting. From the available 74 cases, the 60 cases complied with the inclusion criteria of the study that anticipated in form of participation in the study. In the study, 56 women and 4 men participated. The patient's age ranged from 53 to 80 years with a mean of 65.1 ± 6.7 years. The average body mass index was found to be 27.9 ± 3.4 (Table 1). A nonsignificant difference was observed in the preoperative and operative status. A detailed layout of volunteer recruitment is presented in Figure 2.

**Figure 3:**
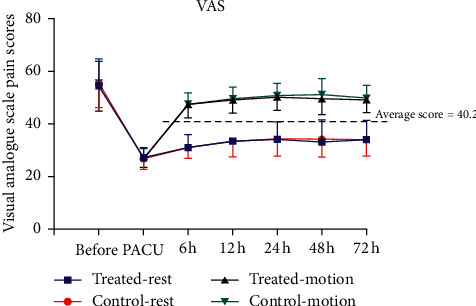
Pain VAS score at rest and on motion (mean and standard deviation) before and after total knee arthroplasty. Time zero is defined as the time immediately after surgery at PACU (postanaesthesia care unit). VAS: visual analogue scale.

**Figure 4:**
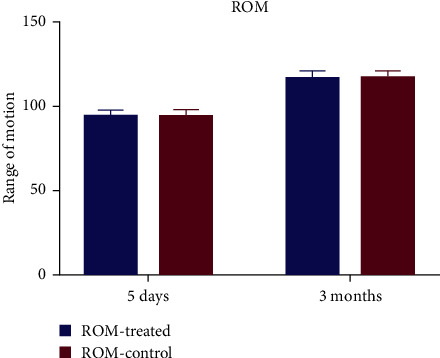
Knee ROM (range of motion) 5 days and 3 months after total knee arthroplasty.

**Figure 5:**
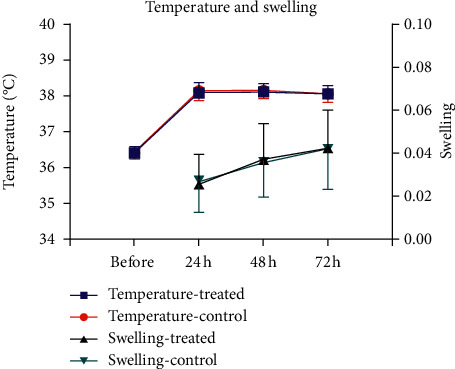
Ratio of the thigh girth at 10 cm proximal from the patella superior border before and following total knee arthroplasty. Skin temperature of the thigh at the patella superior border before and following total knee arthroplasty.

No notable difference in the ratings of the pain during rest and motion was observed (Figure 3) at a certain duration after treatment (Table 2). No statistically relevant variations in secondary results were observed between the two knees (range of movement (Figure 4), clinical score (Figure 1), and swelling of the thigh and thigh surface temperature at either postoperative moment (Figure 5 and Table 2).

There was also no distinction among the two knees on day 1 of surgery and the complete discharge (Table 2). Patients were ineffectual to discern a gap in physical healing among their knees on postoperative day 3 or at 3 months of investigation (Table 3). No indicative deep vein thrombosis or pulmonary embolism and infection at the surgical site were observed for a short duration (3 months) of investigation. The 3 patients obtained transfusion, whereas 19 patients experienced vomiting.

## 4. Discussion

The objective of this research was to examine if corticosteroids are effective and safe as a part of multimodal periarticular combination injections in patients receiving concurrent bilateral complete knee arthroplasty. The groups were comparable at the time of recruitment. Pain scores at resting or in movement, range of movement, clinical values, and complications associated did not vary among the two knees on the third day after surgery and even the three-month treatment period.

Betamethasone, the medication used in this intervention, is one of the long-acting corticosteroids (“half-life = 36–54 min”). It is used for a number of diseases including rheumatic disorders such as rheumatoid arthritis and systemic lupus erythematosus. To date, only three studies have also shown that betamethasone decreases pain sensitivity and supports effective rehabilitation following complete knee arthroplasty [35–37]. More trials may help to validate the utility of longer-acting corticosteroids. Besides the variation in corticosteroid selection, also there is a distinction while selecting the route of administration of this drug. The finding contributes rationale for the use of pneumatic patch compression bandage added following surgery for the management of pain against surgical distress [38]. As a result, this procedure led to reduced oedema and internal bleeding due to decreased synthesis of prostaglandins that induce vasodilation.

Perseverance of pain can lead to degenerative changes in the pain-relaying elements of the nervous system. Neuroimaging data shows that chronic pain can lead to improvements in the insula, mid-brain, and cortical areas. When modified, most of these nociceptive components are possible initiators of pain. A surgical intervention, even within a view to alleviating knee pain, can additionally activate the hyperalgesia system [39, 40]. Patients with unilateral cases of complete knee arthroplasty can get benefits from the locally administered periarticular injection. Patients with unilateral cases of total knee arthroplasty can be benefited from locally administered periarticular injections [41]; however, the same dosage form may not provide relief from pain following total knee arthroplasty at about the same time.

While the other potential alternative is to escalate the dosage of corticosteroids, the massive impact could additionally raise the possibility of wound contamination and breakup of the patellar tendon [19, 42]. Conversely, the use of systematic drug administration can help to relieve postsurgery discomfort at locations outside the surgical site [41, 43]. Research findings utilizing systemic corticosteroids are small in number and demand further proof of effectiveness and security.

The merit of the research would be the use of two surgical sites in the same patient. This has enabled us to free up several specific variables that can affect the progression of postoperative inflammation and discomfort. The benefit of this study was that doctors, surgical personnel, patients, physiotherapists, nurses, and data sources stayed ignorant to patients' knee condition for the entirety of the trial. The drawback of the study is the strategy where injection at periarticular space and administration of the intravenous tranexamic acid was made to avoid postoperative squandering of blood, which may have baffled the analysis of the findings [44]. Third, the findings of this analysis utilizing betamethasone might not have been appropriate for other corticosteroids. There has been debate as to if the combination can be administered to the extensor mechanism. The study was performed, though, along with this study [34]. Chia et al. [19] did not indicate an increase in the likelihood of patellar tendon injury.

## 5. Conclusions

In conclusion, the addition of betamethasone to a multimodal mix periarticular injection, including local anaesthetic, NSAIDs, opioids, and epinephrine, demonstrated no additional local effectiveness. Future research can examine the preference and route of application of corticosteroids.

## Figures and Tables

**Table 1:** Baseline characteristics.

	Age (years)	Female/male	Height (cm)	Weight (kg)	BMI (kg/m^2^)
Mean	65.1	F: 56	159.3	70.83	27.90
SD	6.8	M: 4	5.4	9.26	3.47

**Table 2:** Comparison of primary and secondary outcomes.

	Treated		Control		*t*-statistic	*P* value
	Mean	SD	Mean	SD		
Pain before operation	54.3	9.4	55.4	9.3	−1.277	0.207
Pain at postanaesthesia care unit	27.2	3.8	26.7	4.1	0.864	0.391
Rest pain 6 h after operation	31.0	4.9	31.0	4.1	−0.086	0.932
Rest pain 12 h after operation	33.3	5.3	33.1	5.5	0.465	0.643
Rest pain 24 h after operation	34.2	6.5	34.3	6.5	−0.261	0.795
Rest pain 48 h after operation	33.1	8.4	34.1	6.7	−1.609	0.113
Rest pain 72 h after operation	34	7.4	34	6.23	0	1
Motion pain 6 h after operation	47.4	5.1	47.5	4.4	−0.21	0.835
Motion pain 12 h after operation	49.1	5.0	49.6	4.4	−0.704	0.484
Motion pain 24 h after operation	50.25	4.994	50.83	4.618	−0.943	0.35
Motion pain 48 h after operation	**49.6**	**6.1**	**51.1**	**5.9**	−**2.809**	**0.007**
Motion pain 72 h after operation	49.3	4.9	49.8	4.8	−0.925	0.359
Thigh skin temperature (°C) before operation	36.4	0.18	36.5	0.20	−1.876	0.066
Thigh skin temperature (°C) 24 h after operation	38.1	0.27	38.2	0.29	−2.22	0.03
Thigh skin temperature (°C) 48 h after operation	38.1	0.22	38.2	0.26	−2.367	0.021
Thigh skin temperature (°C) 72 h after operation	38.1	0.24	38.1	0.22	−1.867	0.067
Thigh circumference before operation (cm)	41.20	3.68	41.08	3.68	1.45	0.152
Thigh circumference (cm) 24 h after operation	42.26	3.76	42.17	3.64	1.096	0.277
Thigh circumference (cm) 48 h after operation	42.72	3.68	42.52	3.62	2.429	0.018
Thigh circumference (cm) 72 h after operation	42.92	3.58	42.78	3.63	1.756	0.084
Ratio of the thigh girth 24 h after operation	0.025	0.014	0.027	0.014	−0.779	0.439
Ratio of the thigh girth 48 h after operation	0.037	0.017	0.036	0.016	0.826	0.412
Ratio of the thigh girth 72 h after operation	0.042	0.018	0.042	0.019	0.199	0.843
Clinical score before operation	35.4	3.6	36.0	3.2	−1.415	0.162
Clinical score 3 months after operation	89.2	3.8	89.0	3.6	0.379	0.706
Range of motion (°) 5 days after operation	94.4	3.6	94.6	3.5	−0.244	0.808
Range of motion (°) 3 months after operation	116.5	4.8	116.8	4.7	−0.444	0.659
Drainage 1 day after operation (mL)	187.5	114.6	166.6	112.4	1.273	0.208
Total drainage (mL)	327.8	133.7	306.0	122.7	1.321	0.192
Tourniquet time (min)	87.0	12.2	88.2	13.5	−0.873	0.230

**Table 3:** Patient self-assessment 3 days after surgery.

Self-assessment at 3 days after surgery				
	Prefer steroids (+) side	Prefer steroids (−) side		
	Left side steroids (+)	19	10	
	Right side steroids (+)	17	14	
				60
	*P* = 0.399 (chi-square = 0.712)			

Self-assessment at 3 months after surgery				
	Prefer steroids (+) side	Prefer steroids (−) side		
	Left side steroids (+)	18	11	
	Right side steroids (+)	20	13	
				60
	*P* = 0.113 (chi-square = 0.514)			

## Data Availability

All the data generated or analyzed during this study are included in this published article.

## References

[1] Insall J. N., Binazzi R., Soudry M., Mestriner L. A. (1985). Total knee arthroplasty. *Clinical Orthopaedics and Related Research*.

[2] Hasegawa M., Tone S., Naito Y., Wakabayashi H., Sudo A. (2019). Prevalence of persistent pain after total knee arthroplasty and the impact of neuropathic pain. *The Journal of Knee Surgery*.

[3] Lavie L. G., Fox M. P., Dasa V. (2016). Overview of total knee arthroplasty and modern pain control strategies. *Current Pain and Headache Reports*.

[4] Burns L. C., Ritvo S. E., Ferguson M. K., Clarke H., Seltzer Z., Katz J. (2015). Pain catastrophizing as a risk factor for chronic pain after total knee arthroplasty: a systematic review. *Journal of Pain Research*.

[5] Alattas S. A., Smith T., Bhatti M., Wilson-Nunn D., Donell S. (2017). Greater pre-operative anxiety, pain and poorer function predict a worse outcome of a total knee arthroplasty. *Knee Surgery, Sports Traumatology, Arthroscopy*.

[6] Stone O. D., Duckworth A. D., Curran D. P., Ballantyne J. A., Brenkel I. J. (2017). Severe arthritis predicts greater improvements in function following total knee arthroplasty. *Knee Surgery, Sports Traumatology, Arthroscopy*.

[7] Singh J. A., Jensen M. R., Harmsen W. S., Gabriel S. E., Lewallen D. G. (2011). Cardiac and thromboembolic complications and mortality in patients undergoing total hip and total knee arthroplasty. *Annals of the Rheumatic Diseases*.

[8] Joshi N., Pidemunt G., Carrera L., Navarro-Quilis A. (2005). Stress fracture of the femoral neck as a complication of total knee arthroplasty. *The Journal of Arthroplasty*.

[9] Palmer A. J. R., Rodríguez-Merchán E. C., Rodríguez-Merchán E. C., Oussedik S. (2015). Acute pain management in total knee arthroplasty. *Total Knee Arthroplasty: A Comprehensive Guide*.

[10] Chou R., Gordon D. B., de Leon-Casasola O. A. (2016). Management of postoperative pain: a clinical practice guideline from the American pain society, the American society of regional anesthesia and pain medicine, and the American society of anesthesiologists’ committee on regional anesthesia, executive committee, and administrative council. *The Journal of Pain*.

[11] Goodman S. M., Springer B., Guyatt G. (2017). 2017 American College of rheumatology/American association of hip and knee surgeons guideline for the perioperative management of antirheumatic medication in patients with rheumatic diseases undergoing elective total hip or total knee arthroplasty. *The Journal of Arthroplasty*.

[12] Fischer H. B. J., Simanski C. J. P., Sharp C. (2008). A procedure-specific systematic review and consensus recommendations for postoperative analgesia following total knee arthroplasty. *Anaesthesia*.

[13] Society K. K. (2012). Guidelines for the management of postoperative pain after total knee arthroplasty. *Knee Surgery & Related Research*.

[14] Ikeuchi M., Kamimoto Y., Izumi M. (2014). Effects of dexamethasone on local infiltration analgesia in total knee arthroplasty: a randomized controlled trial. *Knee Surgery, Sports Traumatology, Arthroscopy*.

[15] Klement M. R., Luzzi A. J., Siddiqi A., Valichka K., Sharkey P. F. (2019). Intra-articular corticosteroid injection following total knee arthroplasty: is it effective?. *The Journal of Arthroplasty*.

[16] Mills E. S., Elman M. B., Foran J. R. H. (2018). The risk of acute infection following intra-articular corticosteroid injection into a pre-existing total knee arthroplasty. *The Journal of Arthroplasty*.

[17] Samona J., Cook C., Krupa K. (2017). Effect of intraoperative dexamethasone on pain scores and narcotic consumption in patients undergoing total knee arthroplasty. *Orthopaedic Surgery*.

[18] Zhao J., Davis S. P. (2019). An integrative review of multimodal pain management on patient recovery after total hip and knee arthroplasty. *International Journal of Nursing Studies*.

[19] Chia S. K., Wernecke G. C., Harris I. A., Bohm M. T., Chen D. B., Macdessi S. J. (2013). Peri-articular steroid injection in total knee arthroplasty: a prospective, double blinded, randomized controlled trial. *The Journal of Arthroplasty*.

[20] Christensen C. P., Jacobs C. A., Jennings H. R. (2009). Effect of periarticular corticosteroid injections during total knee arthroplasty. *The Journal of Bone & Joint Surgery*.

[21] Marsland D., Mumith A., Barlow I. W. (2014). Systematic review: the safety of intra-articular corticosteroid injection prior to total knee arthroplasty. *The Knee*.

[22] Wallace S. S., Bechtold D., Sassoon A. (2017). Periprosthetic fractures of the distal femur after total knee arthroplasty : plate versus nail fixation. *Orthopaedics & Traumatology: Surgery & Research*.

[23] Kwon S. K., Yang I. H., Bai S. J., Han C. D. (2014). Periarticular injection with corticosteroid has an additional pain management effect in total knee arthroplasty. *Yonsei Medical Journal*.

[24] Fu D., Li G., Chen K., Zeng H., Zhang X., Cai Z. (2013). Comparison of clinical outcome between simultaneous-bilateral and staged-bilateral total knee arthroplasty: a systematic review of retrospective studies. *The Journal of Arthroplasty*.

[25] Leitch K. K., Dalgorf D., Borkhoff C. M., Kreder H. J. (2005). Bilateral total knee arthroplasty--staged or simultaneous? Ontario’s orthopedic surgeons reply. *Canadian Journal of Surgery-Journal canadien de chirurgie*.

[26] Malahias M.-A., Gu A., Adriani M., Addona J. L., Alexiades M. M., Sculco P. K. (2019). Comparing the safety and outcome of simultaneous and staged bilateral total knee arthroplasty in contemporary practice: a systematic review of the literature. *The Journal of Arthroplasty*.

[27] Kim M.-H., Nahm F. S., Kim T. K., Chang M. J., Do S.-H. (2014). Comparison of postoperative pain in the first and second knee in staged bilateral total knee arthroplasty: clinical evidence of enhanced pain sensitivity after surgical injury. *Pain*.

[28] Moore A. J., Gooberman‐Hill R. (2020). Why don’t patients seek help for chronic post‐surgical pain after knee replacement? A qualitative investigation. *Health Expectations*.

[29] Larsen D. B., Laursen M., Edwards R. R., Simonsen O., Arendt-Nielsen L., Petersen K. K. (2021). The combination of preoperative pain, conditioned pain modulation, and pain catastrophizing predicts postoperative pain 12 Months after total knee arthroplasty. *Pain Medicine-Malden, Mass*.

[30] Hardaker W. T., Ogden W. S., Musgrave R. E., Goldner J. L. (1978). Simultaneous and staged bilateral total knee arthroplasty. *The Journal of Bone and Joint Surgery. American Volume*.

[31] Alghadir A. H., Iqbal Z. A., Anwer S., Anwar D. (2020). Comparison of simultaneous bilateral versus unilateral total knee replacement on pain levels and functional recovery. *BMC Musculoskeletal Disorders*.

[32] Hussain N., Chien T., Hussain F. (2013). Simultaneous versus staged bilateral total knee arthroplasty. *HSS Journal*.

[33] Huang G. F., Zhang H. X., Zhang T. F., Yu F. (2006). Time-dependent analgesic effect of electroacupuncture at Jiaji acupoint in patients with lumbar disc herniation and its intervention on related factors of plasma. *Chinese Journal of Clinical Rehabilitation*.

[34] Tsukada S., Wakui M., Hoshino A. (2014). Postoperative epidural analgesia compared with intraoperative periarticular injection for pain control following total knee arthroplasty under spinal anesthesia. *Journal of Bone and Joint Surgery*.

[35] Fu P., Wu Y., Wu H., Li X., Qian Q., Zhu Y. (2009). Efficacy of intra-articular cocktail analgesic injection in total knee arthroplasty-a randomized controlled trial. *The Knee*.

[36] Liu P., Guo W. S., Zhang Q. D., Wang W. G., Zhang Y., Wang Y. Y. (2019). [Efficacy and safety of compound betamethasone cocktail therapy in primary unilateral unicompartmental knee arthroplasty]. *Zhonghua Yi Xue Za Zhi*.

[37] Moráis S., Ortega-Andreu M., Rodríguez-Merchán E. C. (2014). Blood transfusion after primary total knee arthroplasty can be significantly minimised through a multimodal blood-loss prevention approach. *International Orthopaedics*.

[38] Migliorini F., Maffulli N., Aretini P. (2021). Impact of tourniquet during knee arthroplasty: a bayesian network meta-analysis of peri-operative outcomes. *Archives of Orthopaedic and Trauma Surgery*.

[39] Huang H., Tanner J., Parvataneni H. (2018). Impact of total knee arthroplasty with general anesthesia on brain networks: cognitive efficiency and ventricular volume predict functional connectivity decline in older adults. *Journal of Alzheimer’s Disease*.

[40] Lan F., Lin G., Cao G. (2020). Altered intrinsic brain activity and functional connectivity before and after knee arthroplasty in the elderly: a resting-state fMRI study. *Frontiers in Neurology*.

[41] Zhou G., Ma L., Jing J., Jiang H. (2018). A meta-analysis of dexamethasone for pain management in patients with total knee arthroplasty. *Medicine*.

[42] Nöth U., Trojanowski M., Reichert J. C., Rolf O., Rackwitz L. (2016). Rupturen der Patellarsehne nach Kniegelenkersatz. *Der Orthopäde*.

[43] Mohammad H. R., Hamilton T. W., Strickland L., Trivella M., Murray D., Pandit H. (2018). Perioperative adjuvant corticosteroids for postoperative analgesia in knee arthroplasty. *Acta Orthopaedica*.

[44] Prakash J., Seon J. K., Song E. K., Lee D. H., Yang H. Y., Jin C. (2018). Is combined administration of tranexamic acid better than both intravenous and topical regimes for total loss, hidden loss and post-operative swelling? A randomized control trial. *Indian Journal of Orthopaedics*.

